# Research on non-cohesive jet formed by Zr-based amorphous alloys

**DOI:** 10.1038/s41598-023-30836-0

**Published:** 2023-03-13

**Authors:** Jin Shi, Zhengxiang Huang, Xudong Zu, Qiangqiang Xiao, Yuting Wang

**Affiliations:** 1 School of Mechanical Engineering, Nanjing University of Science and Technology, Nanjing, 210094 People’s Republic of China; 2 College of Field Engineering, Army Engineering University of PLA, Nanjing, 210007 People’s Republic of China

**Keywords:** Mechanical engineering, Metals and alloys, Computational methods

## Abstract

The shaped charge jet formation of a Zr-based amorphous alloy and the applicability of different numerical algorithms to describe the jet formed were experimentally and numerically investigated. X-ray experiments were performed to study jet characteristics. The numerical results for the Zr-based amorphous alloy jet formed via the Euler and smooth particle hydrodynamics (SPH) algorithms were compared and analyzed using the Autodyn hydrocode. Particle motion was examined based on material properties. The Zr-based amorphous alloy formed a noncohesive jet driven by an 8701 explosive. Both the Euler and SPH algorithms achieved high accuracy for the determination of jet velocity. When the improved Johnson-Holmquist constitutive model (JH-2) was used, numerical results confirmed the model’s suitability for the Zr-based amorphous alloy. The Euler algorithm effectively reflected jet shape within a short computing time, whereas the SPH algorithm was highly suitable for showing the shape of the jet tail within a long computing time. In the 3D Euler model, the flared jet mouth indicated radial particle dispersion; however, in the 2D model, particle dispersion in the head was directly observed by using the JH-2 material model. The brittle fracture of the material reduced the proportion of particles near the liner apex forming a jet. Furthermore, a new method in which stagnation pressure was used to predict jet formation and its coherence was proposed since the collapse angle was difficult to obtain.

## Introduction

Theoretical studies and experiments have shown that the penetration ability of jets is closely related to their mechanical properties and cohesiveness. Researchers^1–3^ have found that the sound speed of a material and the collapsing velocity of the liner are related to the cohesiveness of the jet. The stability of the jet and other factors directly affect the penetration ability of the shaped charge^4–7^. Therefore, multiple types of material liners have been studied to achieve a good penetration ability. Copper, tantalum, zirconium (Zr), depleted uranium, and other metals have been tested^8–10^. Numerous types of alloys, active materials, and nonmetal liners have been studied^11–18^. With the rapid development of material science, various new materials have been gradually developed and applied to practical engineering. Among these materials, amorphous alloy materials are favored due to their unique deformation mechanism and excellent mechanical properties, such as high strength and toughness, and are widely used in mechanical, aerospace, and military fields^19,20^. Due to the ultrahigh strength and self-sharpening characteristics of these materials, amorphous alloy armor-piercing projectiles with greatly improved penetration power and target after-effects have been developed^21–23^. Different amorphous composite materials are also widely used for military protection due to the high strength of amorphous alloys^24^. In contrast to amorphous alloy materials, which are being rapidly developed for armor protection and kinetic energy projectiles, there have been relatively few studies on their application as a shaped charge liner^25,26^.

In this study, the jet formation characteristics of a Zr-based amorphous alloy liner were compared and analyzed via experiments and numerical simulations. The accuracy and applicability of the Euler and smooth particle hydrodynamics (SPH) algorithms to the Zr-based amorphous alloy jet were examined and verified. The Zr-based amorphous alloy and Zr jets were numerically compared, and the differences between the liner crushing and jet forming behaviors of the two materials were analyzed. A method for predicting jet forming conditions on the basis of collision pressure is proposed to avoid using the collapse angle, which is difficult to accurately obtain. The results can not only provide a reference for the prediction of jet formation and coherency but also broaden the application value of Zr-based amorphous alloy liners in engineering.

## Research methodology and analysis of results

### Jet forming experimental setup

**Figure 1:**
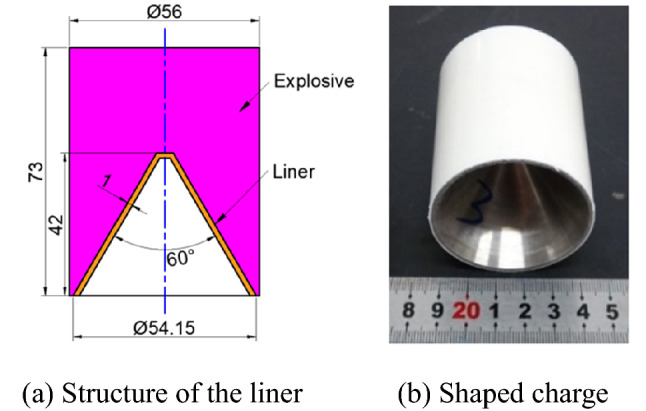
Shaped charge used for the X-ray experiment.

**Figure 2:**
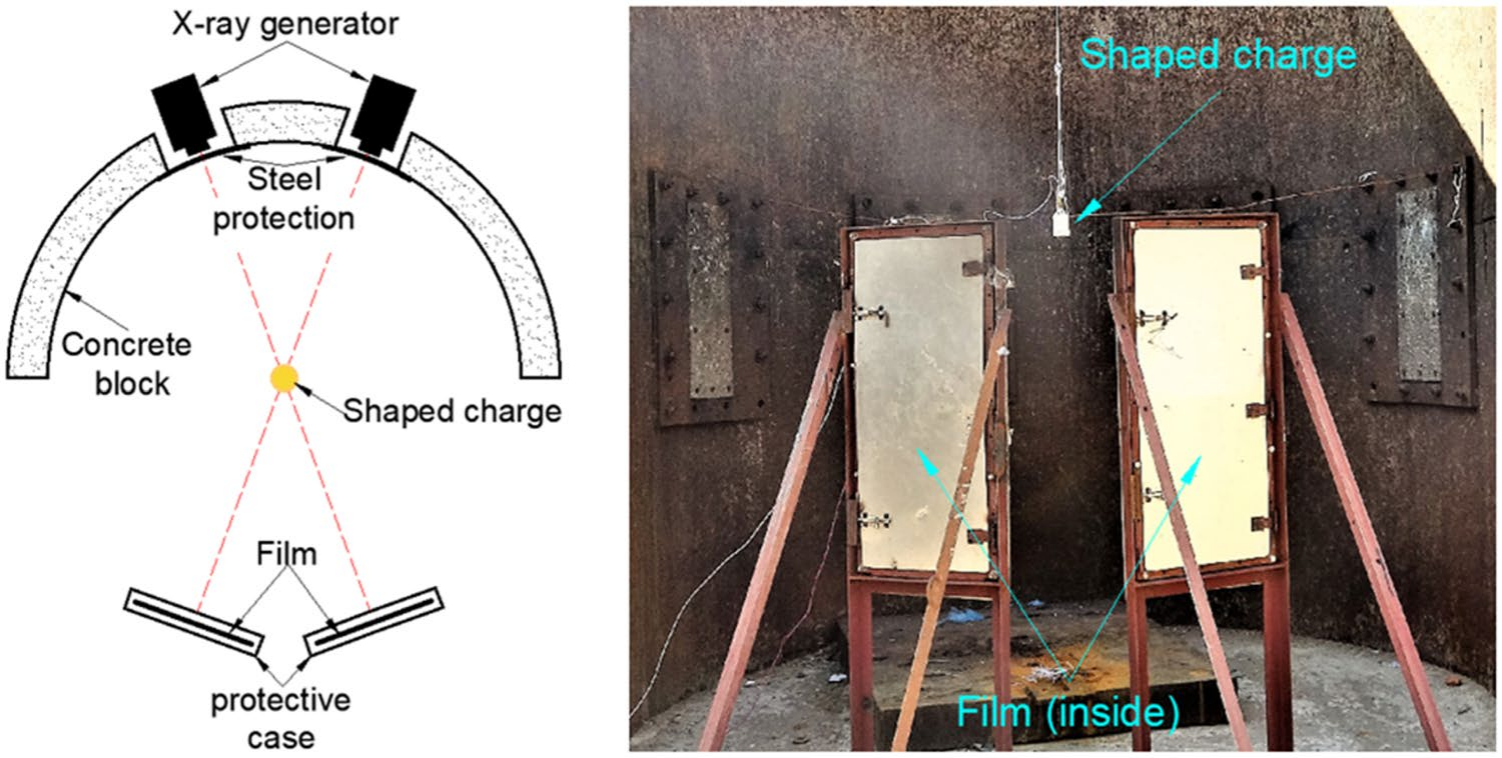
Schematic illustration of the X-ray experiment.

To examine the jet performance of the Zr-based amorphous alloy liner under an explosive drive, an X-ray test was performed. The nominal composition of the liner material is Zr_41.2_Ti_13.8_Cu_12.5_Ni_10_Be_22.5_ (Vit1). The density of the liner is 6.11 g/cm^3^, and the shaped charge used has an outer diameter of Φ56 mm. The 8701 explosive has a density of 1.72 g/cm^3^. The detailed parameters of the materials are provided in the appendix. The structures of the liner and the shaped charge are shown in Fig. 1. The X-ray experimental setup is shown in Fig. 2, which shows that the shaped charge is suspended at the intersection of the two X-ray generators.

### X-ray results and analysis

Three experiments were performed, and all formed noncohesive jets. The jet morphologies at 30, 40, and 60 μs after detonation were obtained.

**Figure 3:**
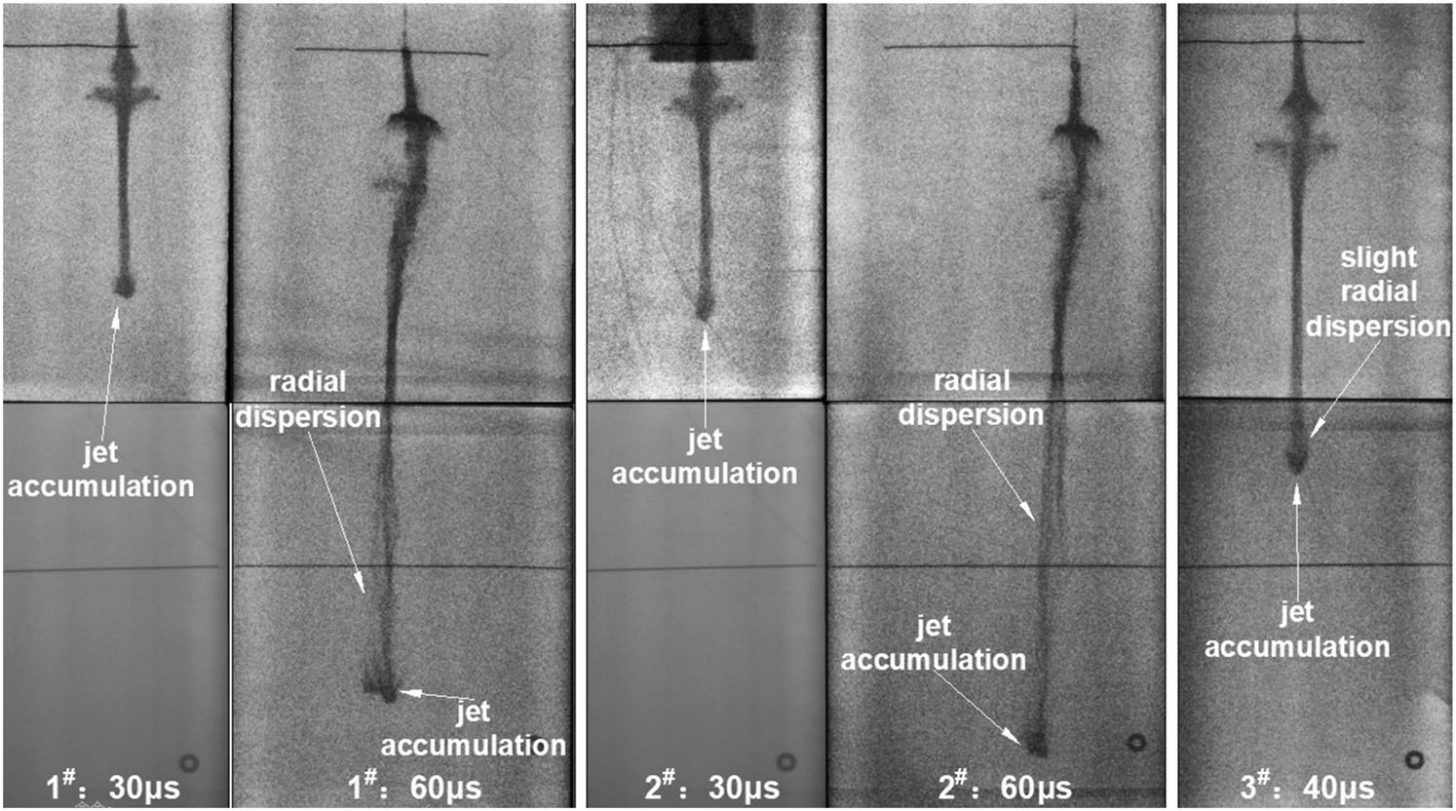
Jet X-ray image.

**Figure 4:**
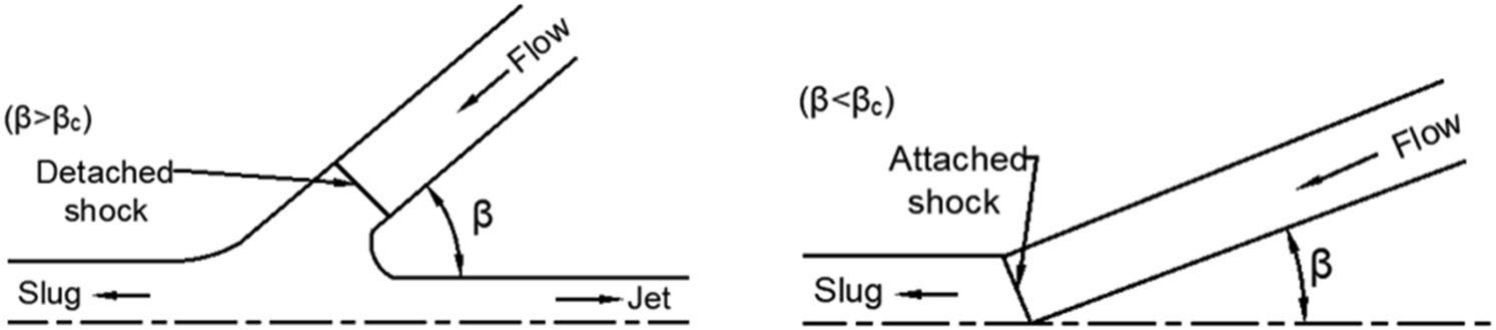
Flow configuration in the supersonic regime^1^.

The X-ray results (Fig. 3) showed that at 30 μs after the charge was detonated, a good straight jet formed. The diameter of the jet body was basically consistent, while the head diameter was slightly larger than that of the body due to particle accumulation. At 40 μs, the jet head continued to expand with a slight radial dispersion of its neck. Then, a markedly noncohesive jet was formed at 60 μs. According to existing research^1–3^, a supersonic flow part of the jet (flow velocity > material’s sound speed) produces a detached shock when the collapse angle is larger than the critical angle ($\beta > \beta_{c}$), as shown in Fig. 4. This results in the radial motion of the particles, which forms a noncohesive jet. The jet accumulation phenomenon of the head at 30 μs may also imply that the particles already followed a radial dispersion trend.

The jet head further scattered radially under air resistance disturbance with time, seriously dispersing the front of the jet radially and forming a cavity at the neck position, as shown by the shape of the jet at 60 μs in Fig. 3. The bending deformation of the jet tail caused by the machining error of the liner also gradually increased to a significant degree with stretching of the jet.

In addition, the distribution of jet particles was analyzed through the brightness of the jet. At 30 μs, the particle concentration was consistent, and only the neck position was slightly lower. The difference in particle distribution tended to become obvious with time, and the middle section of the jet showed a certain particle clustering. At 60 μs after initiation, the particle densities at the tail and middle sections of the jet remained higher than that at the jet neck area, but they were significantly lower than that at the previous moment under the influence of radial divergence.

Table 1 shows the relevant jet results from the X-ray experiments. The jet velocity is the average velocity obtained from the three sets of experimental data. The diameter of the jet tip was always larger than that of the tail at different times. Due to the different radial velocities, the difference in the diameters between the jet tip and tail increased rapidly with time. The large radial velocity at the head of the jet and the large axial velocity gradient caused the jet to expand continuously during stretching. Consequently, the jet head appeared like a “trumpet” with a small cone angle.

**Table 1:** Jet experimental results.

Time/μs	Jet length/mm	Jet diameter/mm		Jet axial velocity/m·s^−1^		Jet radial velocity/m·s^−1^	
		Tip	Tail	Tip	Tail	Tip	Tail
30	114	11.83	9.92	7069	671	92	35
40	173	13.52	10.87				
60	300	18.19	12.74				

X-ray experiments can directly demonstrate the jet shape macroscopically, providing direct evidence of jet formation. However, due to a limited number of ray tubes, the available data are relatively constrained and thus insufficient to support an in-depth understanding of the internal mechanism of jet formation. Therefore, numerical simulation is further used to study jet formation to compensate for the limited X-ray experiments.

### Numerical model and operation

The Autodyn hydrocode (Version 19.0) was used to calculate jet formation and examine the accuracy and feasibility of different algorithms by numerically simulating the Vit1 jet, providing a reference for the numerical calculation of noncohesive jets. Detailed descriptions of the material models and parameters used in the simulation are provided in section the “Appendix” (in Supplementary Information).

**Figure 5:**
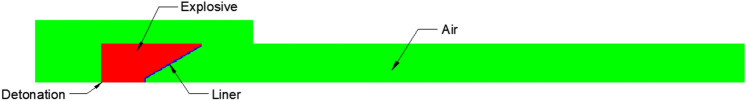
2D axial symmetry numerical model.

The Euler algorithm can effectively solve large deformation problems in explosions because of the absence of grid intersections. This algorithm overcomes the calculation problem of the Lagrange algorithm due to grid distortion. A 2D axial symmetry model was established to improve the efficiency, as shown in Fig. 5. The detonation was set at the center of the end face. The mesh size of the charge and the jet channel was 0.1 mm × 0.1 mm. The air domain was approximately 520 mm × 90 mm, which sets the “flow_out” boundary. The width of the jet channel’s air domain was reduced to the same value as the diameter of the charge to improve the operation efficiency.

**Figure 6:**
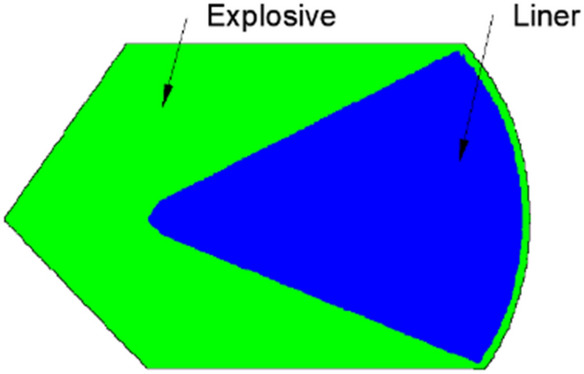
SPH simulation model.

The SPH is a meshless algorithm based on Lagrange and uses the mass, energy, and momentum of particles to form discrete computational fields^27^. The deformation of materials is not dependent on the size and distribution of the mesh but is naturally expressed by particles. The algorithm can improve the interface problem between the mesh and material in the Euler framework and is suitable for solving dynamic large-deformation problems. Therefore, SPH is also used for the Vit1 jet forming calculation to verify whether the algorithm is suitable for reflecting the particle state of noncohesive jets. The 3D Lagrange model of the shaped charge established in ANSYS (Version 19.0) was imported into Autodyn. Then, the Lagrange model was converted into the SPH model. The 1/4 shaped charge model was built to improve efficiency (Fig. 6).

Particle size (also called the smoothing length) has a significant impact on the accuracy of the calculation results and ensues that the same particle size between parts can reduce the calculation error. Thus, particle sizes of 0.1, 0.2, 0.3, 0.4 and 0.5 were considered and compared.

There are some items to consider in the jet forming simulation from the two algorithms:

● Timestep is an important value used to maintain a stable calculation. Numerous trials have shown that the timestep magnitude order in jet forming simulations should be maintained between 10^–6^ and 10^–5^. When the timestep magnitude order is less than 10^–6^, the simulation runs erratically. In addition, the minimum timestep value is usually multiplied by a safety factor to ensure stability. The parameters involved in the simulation are listed in Table 2.

**Table 2:** Control parameters of the simulation.

Maximum timestep	Minimum timestep	Safety factor	Quadratic viscosity	Linear viscosity
1.0 × 10^8^	1.0 × 10^–6^	0.67	1.0	2.0

● Instability may occur during the simulation; this instability is shown by the appearance of abnormally high velocity particles at the boundary. Filling the mesh of these particles with voids in the Euler model or deleting these particles in the SPH model solves this instability problem and enables the simulation to run smoothly. The domain of particles also becomes clear and is easy to track after removing the problem particles.

### Numerical results and analysis

**Figure 7:**
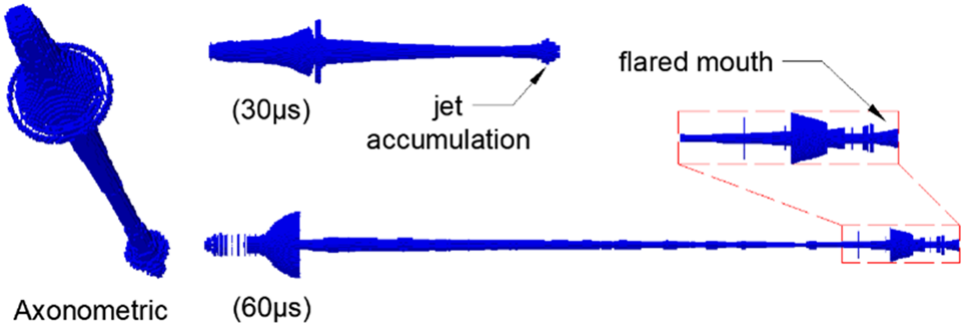
3D results under the Euler algorithm.

For comparison with the actual jet obtained from the X-ray experiments, the 2D results using the Euler algorithm were converted into 3D models, as shown in Fig. 7.

**Figure 8:**
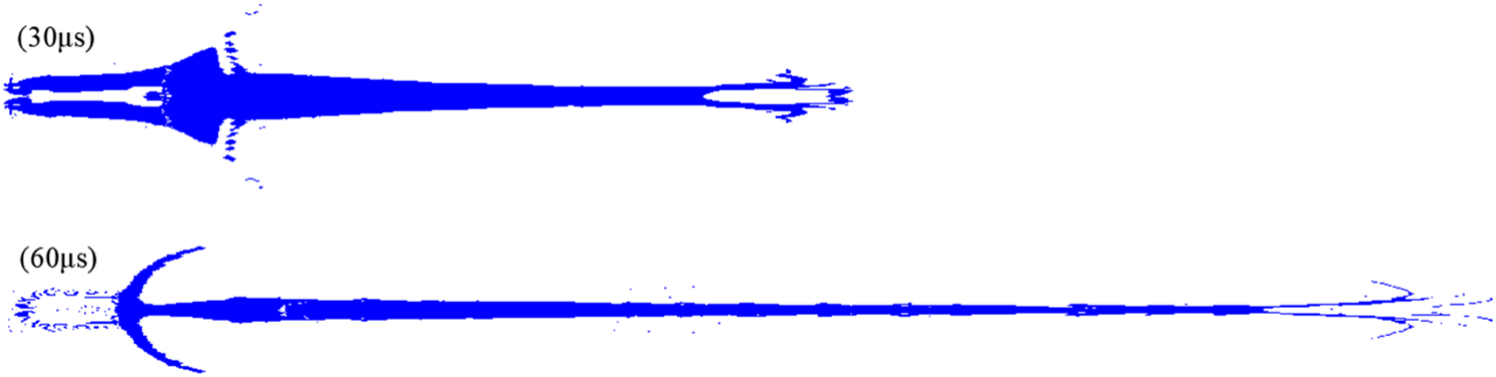
2D jet morphology under the Euler algorithm.

The jet shape obtained by simulation at 30 μs is generally consistent with the experimental results. The jet accumulation phenomenon in the head is also effectively modeled. The significant difference from the actual jet lies only in the obvious gradient of the jet diameter from the neck to the tail in the numerical simulation. However, the morphology of the noncohesive jet is gradually distorted with time and cannot directly reflect the radial dispersion. The simulation results at 60 μs show that the jet head has a trumpet shape with the mouth forward, which may indicate the radial dispersion trend of the jet. Figure 8 shows the 2D jet morphology of Vit1 under the Euler algorithm, further demonstrating the internal situation of the jet. Figure 8 shows that at 30 μs, the head of the jet has a cavity. This phenomenon may imply the existence of a hollow domain in the head of the actual jet, which cannot be directly observed in the X-ray image because it is surrounded by outer particles. At 60 μs, the simulation results show that the jet head splits and bends outward, resembling a crater. Although the simulation results at this time are quite different in shape from the actual jet, the radial dispersion of the particles in the head are directly observed. This result is more intuitive than the 3D jet morphology shown in Fig. 7, which is useful for evaluating the cohesiveness of the jet.

Figures 7 and 8 show that within a short calculation time, the 3D model of the calculation results effectively reflects the jet morphology, whereas the 2D model is very useful in understanding the internal situation. Therefore, the calculation results of the 2D model using the Euler algorithm are summarized in Table 3 (“−” indicates that the particles gather inward). A comparison of Tables 1 and 2 shows that the error between the simulation and experimental results is very small, and only the radial velocity at the jet tail is significantly different. Contrary to the actual jet, the diameter of the jet tail decreases with time in the simulation because its radial velocity direction is toward the central axis.

**Table 3:** Simulation results of the 2D/Euler algorithm.

Time/μs	Jet length/mm	Jet diameter/mm		Jet axial velocity/m·s^-1^		Jet radial velocity/m·s^-1^	
		Tip	Tail	Tip	Tail	Tip	Tail
30	106	8.8	7.2	7075	634	109	−19
40	162	9.4	6.4	7060	630	97	−17
60	303	12.8	4.6	7027	619	81	−12

**Figure 9:**
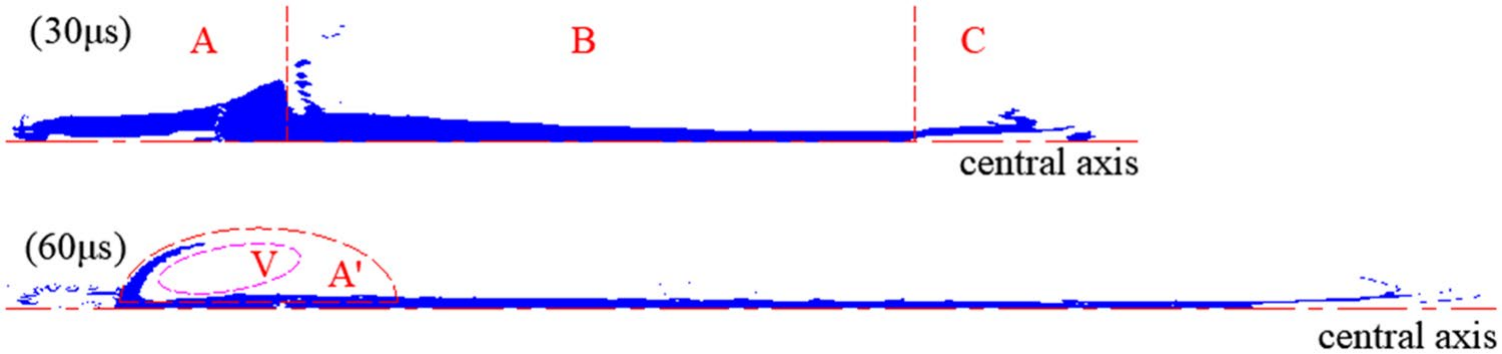
Distribution of radial velocity.

The analysis of the radial velocity of the jet at 30 μs shows that the jet could be roughly divided into three parts along the axial direction, as shown in Fig. 9. Regions A and C of the jet had positive radial velocities, resulting in the radial divergence of the particles in these regions. However, the radial velocity of the jet elements in region B was negative, indicating that the jet diameter decreased gradually with time. In general, the particles in region A should theoretically fill the corresponding region A' from the axial and radial motions. The maximum radial size of the position shown in the V region is often measured to be the effective diameter of the jet tail. However, under the Euler algorithm, no particles were found in this region at this moment, resulting in large errors in the diameter of the jet tail. Furthermore, the calculated radial velocity was opposite that of the actual jets.

**Figure 10:**
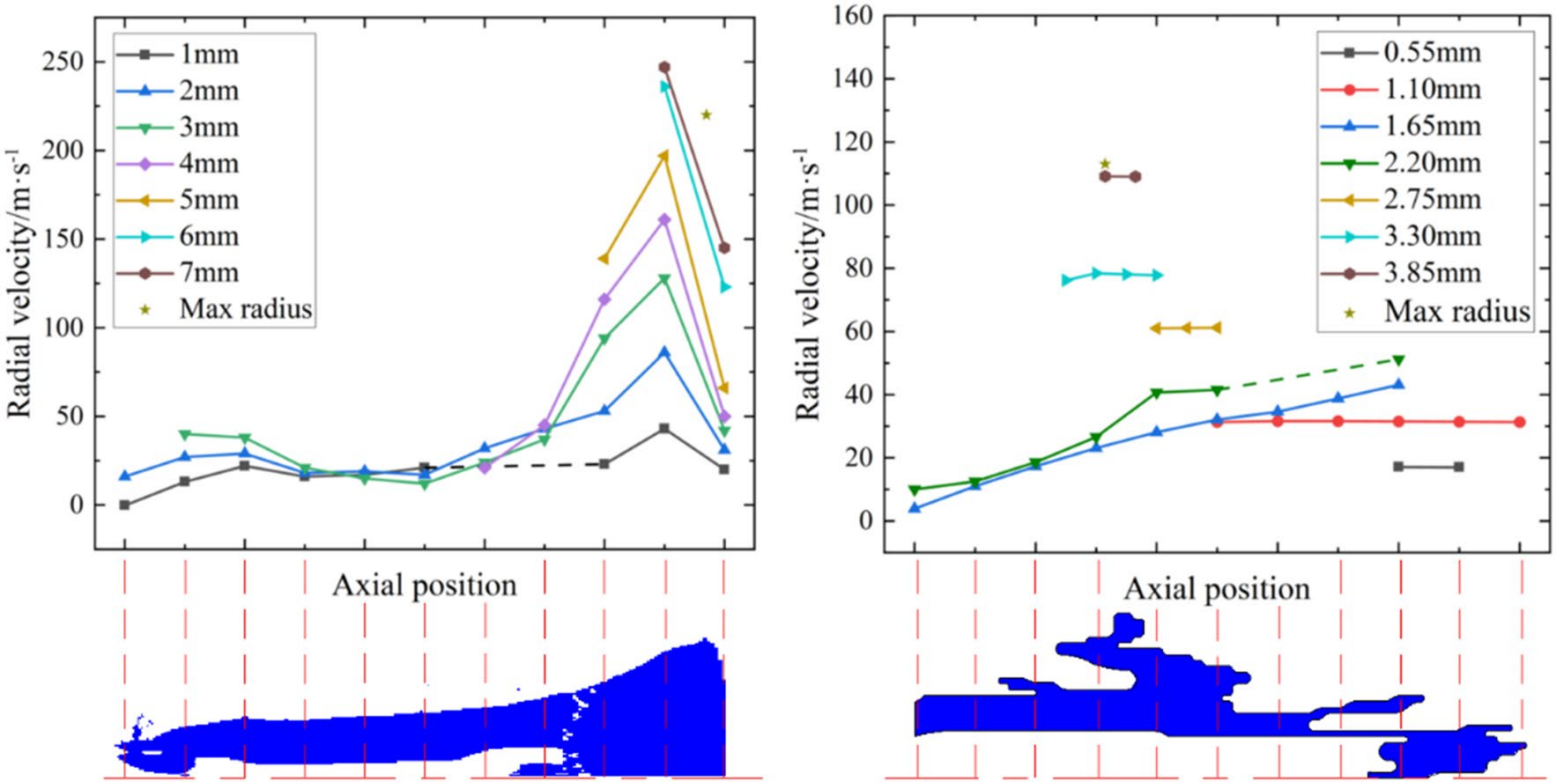
Radial velocities at different positions of the jet tip and tail at 30 μs (Left: radial velocity of region A; Right: radial velocity of region C).

The dotted line in Fig. 10 indicates the cavity or absence of a jet at the relevant position. For the jet tail, the radial velocity of the particles initially increased and then decreased along the direction of motion. In the middle of region A, a larger radius correlated to a lower radial velocity. At the other positions, the radial velocity of the particles at different radii showed the same variation as that of the jet head; specifically, a larger radius correlated to a greater radial velocity. Owing to air disturbance, a greater radial velocity occurred when the particles in the jet head cavity were more forward.

Therefore, the Euler algorithm can effectively simulate the noncohesive jet formed by Vit1 within a relatively short computing time, with a relatively small error in velocity and shape. The Euler algorithm can also reflect the actual movement trend of particles, providing a good reference for studying the collapse deformation of the liner and predicting features for jet formation. However, the ability of the algorithm to characterize particle dispersion decreased significantly with time, and the jet shape distortion worsened. Therefore, the applicability of the SPH algorithm to noncohesive jets with a long computing time was examined.

**Figure 11:**
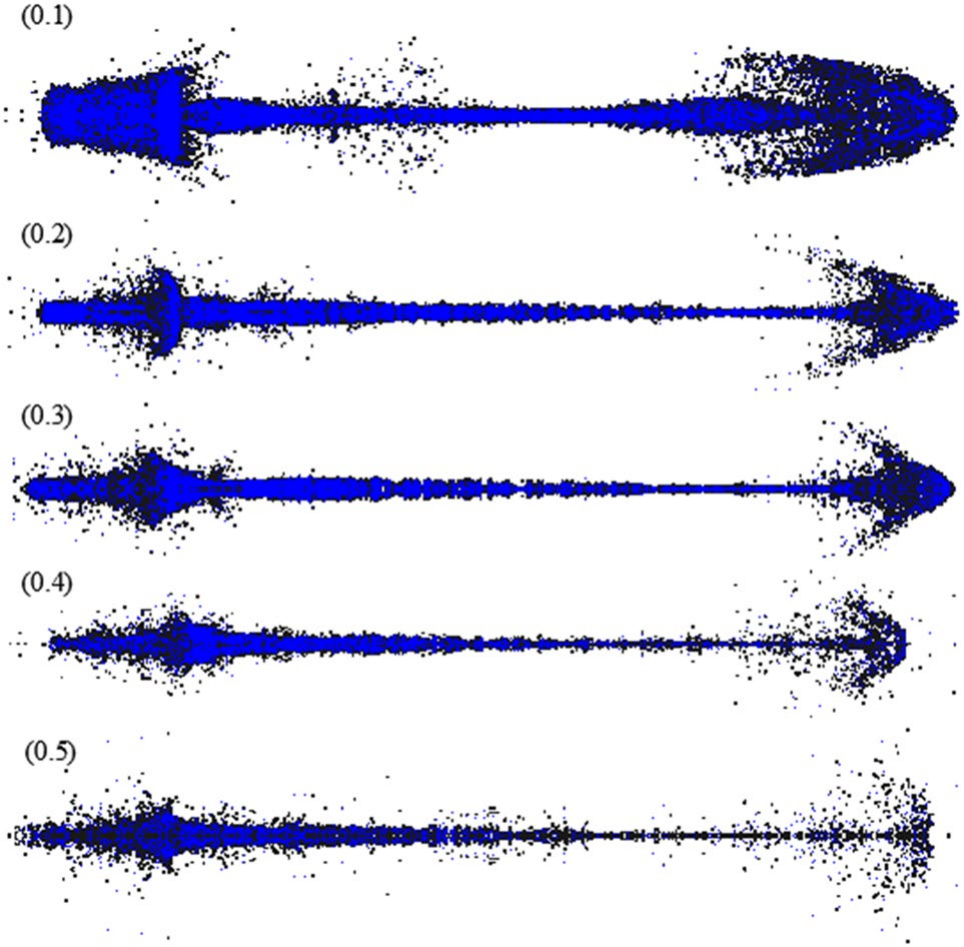
Jet shape of the different particle sizes (60 μs).

Figure 11 shows the significant impact of particle size on jet shape at 60 μs. When the particle size was 0.1 mm, the particles in the jet tip were abundant, and the shape was more clearly characterized. By contrast, the shape of the jet tail showed a high degree of reduction when the particle size increased. However, the head of the jet was nearly arrow-shaped, which was inconsistent with the trumpet shape of the actual jet. Compared with the Euler algorithm, the particle distribution at the tail of the jet at this time was more consistent with the X-ray results, effectively filling the particle vacancy in the V region, as shown in Fig. 9. The jet calculation results are listed in Table 4.

**Table 4:** Calculation results of the different particle sizes at 60 μs.

Particle size/mm	Jet length /mm	Jet diameter/mm		Jet axial velocity/m·s^−1^		Jet radial velocity/m·s^−1^	
		Tip	Tail	Tip	Tail	Tip	Tail
0.1	291	22.6	9.0	7024	724	175	35
0.2	297	20.7	11.3	7073	717	127	72
0.3	298	18.9	13.2	7100	706	106	45
0.4	305	16.8	12.1	7060	690	98	29
0.5	311	16.0	11.8	6961	745	71	27

The influence of particle size on the accuracy of calculating the jet’s radial velocity was particularly obvious. When the particle size was 0.3 and 0.4 mm, the size and velocity of the jet obtained by simulation had a small error compared with the actual results. An obvious particle cluster structure was found at the middle of the jet when the particle size was 0.3 mm, consistent with the X-ray results.

The diameter of the front part of the jet was significantly larger than that of the middle part when the particle size was 0.1 mm, which was consistent with the actual situation. The operation time was abnormally long, and the cost-effectiveness ratio was extremely high at this particle size. Therefore, the preferred particle size was 0.3 mm to preserve the shape of the noncohesive jet to the greatest extent, ensure the calculation accuracy, and improve the efficiency.

In summary, for the Vit1 jet, both the Euler and SPH algorithms could obtain relatively accurate calculation results. To simulate a noncohesive jet, the Euler algorithm can meet the requirements for an accurate calculation when the liner collapses up until the jet is formed. Euler’s 3D model can reflect the jet shape within a short computing time, whereas the 2D model is useful in understanding the internal situation of the jet. Moreover, the flared jet head under the 3D model indicates that the particles have a radial dispersion trend, which may be used to predict the cohesiveness of the jet. The SPH algorithm is suitable for determining the shape of the jet tail at long computation times. However, the shape of the noncohesive jet head is highly distorted under the SPH algorithm, while the radial dispersion phenomenon of the noncohesive jet is directly observed under the Euler algorithm with the use of a suitable material model.

## Comparative discussion

Recent experiments showed that zirconium (Zr) liners of different structures formed a cohesive jet^28^, which was completely different from Zr-based amorphous alloys. Therefore, numerical methods were used to study the crushing and jet forming process of the two liner materials. On the basis of the discussion regarding the applicability of the numerical algorithms in the previous section, the Euler algorithm was used to carry out these simulations. The data in the Autodyn material library were directly used as Zr material parameters, and the simulation model was consistent with the relevant descriptions in section "Research methodology and analysis of results".

### Jet forming

The first 30 μs simulation results of the jet forming of the two liner materials were recorded, as shown in Table 5.

**Table 5:** Comparison of jet shape between Zr and Vit1.

Time/μs	Zr	Vit1
10		
20		
30		

The macroscopic morphologies showed that the deformations of the two materials were similar, except for the obvious cavity at the jet head and tail of Vit1. At 30 μs after initiation, the jet of the two materials had basically formed. Zr formed a cohesive jet, which was consistent with a previous report^28^, while the jet tip of Vit1 showed a significant noncohesive state, with its head diameter increasing significantly. These results are listed in Table 6.

**Table 6:** Simulation results of two materials at 30 μs.

Material	Jet velocity/m·s^-1^		Jet diameter/mm		Jet length/mm
	Tip	Tail	Tip	Tail	
Zr	7453	147	4.2	5.8	118
Vit1	7087	447	8.0	7.2	105

A notable problem is that the density of Zr (6.5 g/cm^3^) is larger than that of Vit1 (6.11 g/cm^3^), and the head velocity of its jet is also relatively larger. However, it is generally believed that a high density will restrict the growth of jet velocity; this is obviously contrary to the simulation results. Therefore, the energy changes in the crushing process of the two kinds of liner material were studied to reveal the causes of the above phenomena.

**Figure 12:**
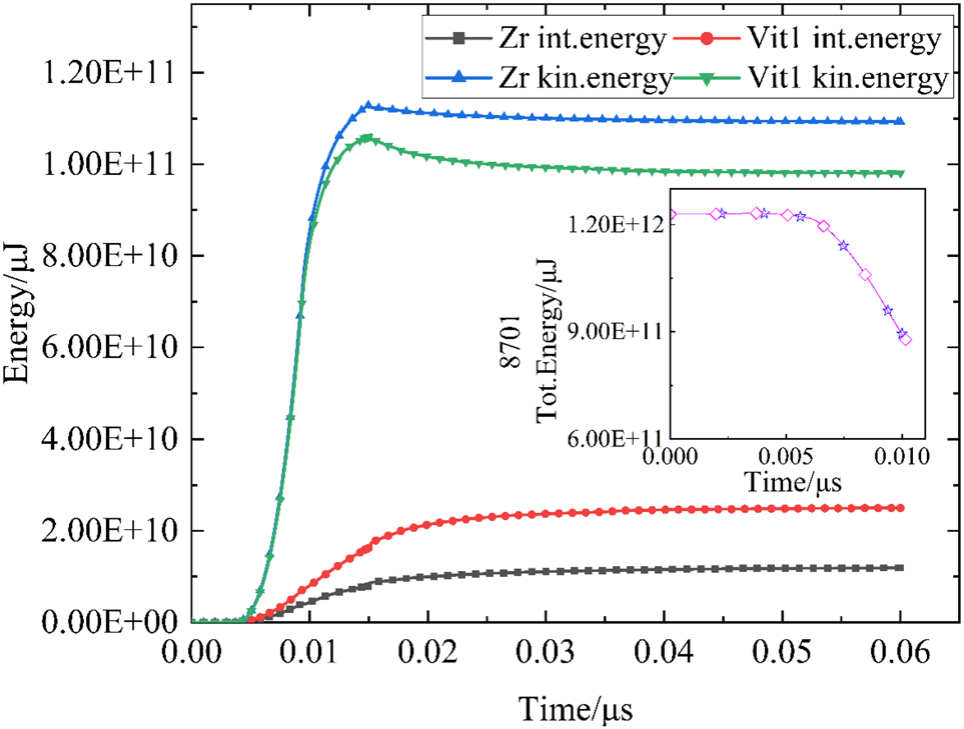
Energy curve of shaped charge.

The energy curve (Fig. 12) indicates that the initial energy and attenuation process of the explosives in the two shaped charges are the same. In the process of the liner collapse of Vit1, its internal energy rapidly increases, and the kinetic energy is low because Vit1 is a reactive material, which mainly relies on the deformation mechanism of the shear transformation zone^29^. The deformation of the shear band leads to a violent adiabatic temperature rise and a rapid increase in internal energy. On the basis of a certain initial charge energy, the increase in the internal energy is bound to reduce the kinetic energy. Therefore, the unique properties of Vit1 cause high internal energy during the brittle fracture of the liner, which inhibits the velocity of particles. Meanwhile, the radial discrete motion of Vit1 jet particles further weakens its axial velocity according to the energy conservation rule.

### Particles in different layers

**Figure 13:**
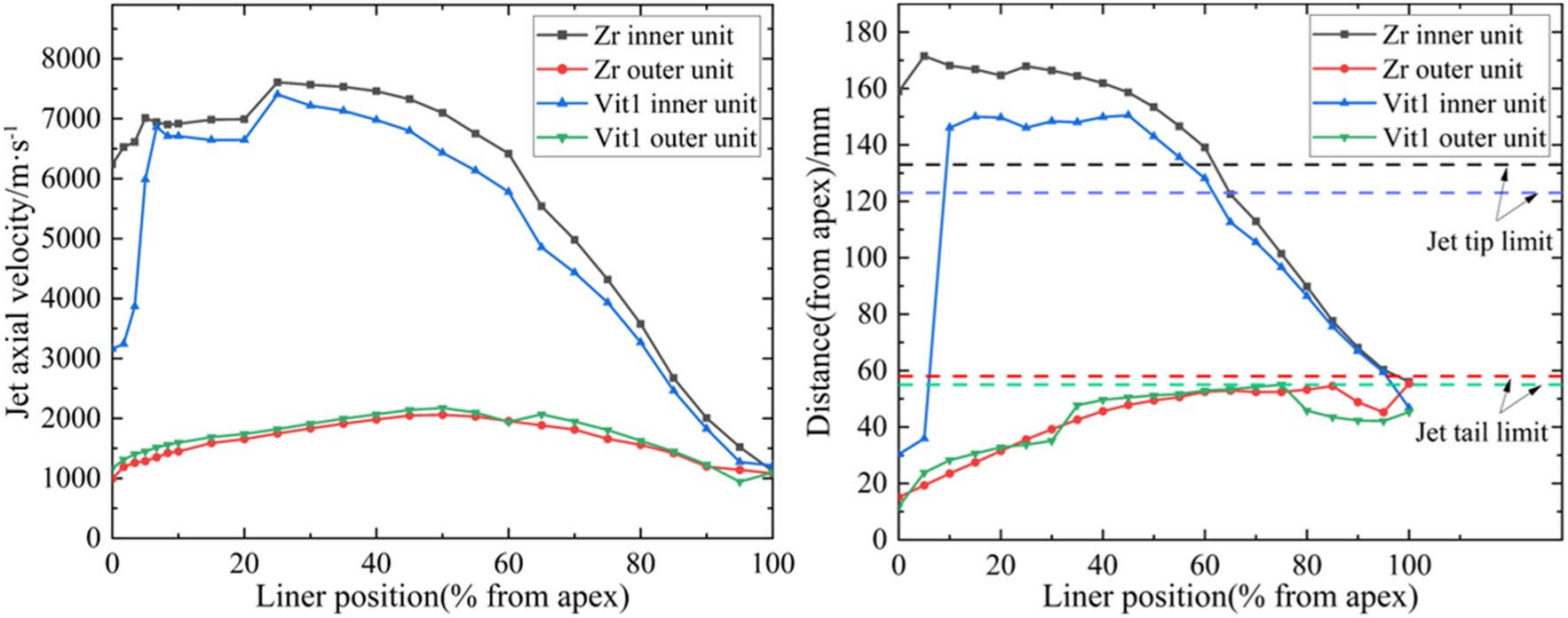
Particle motion characteristics (30 μs).

**Figure 14:**
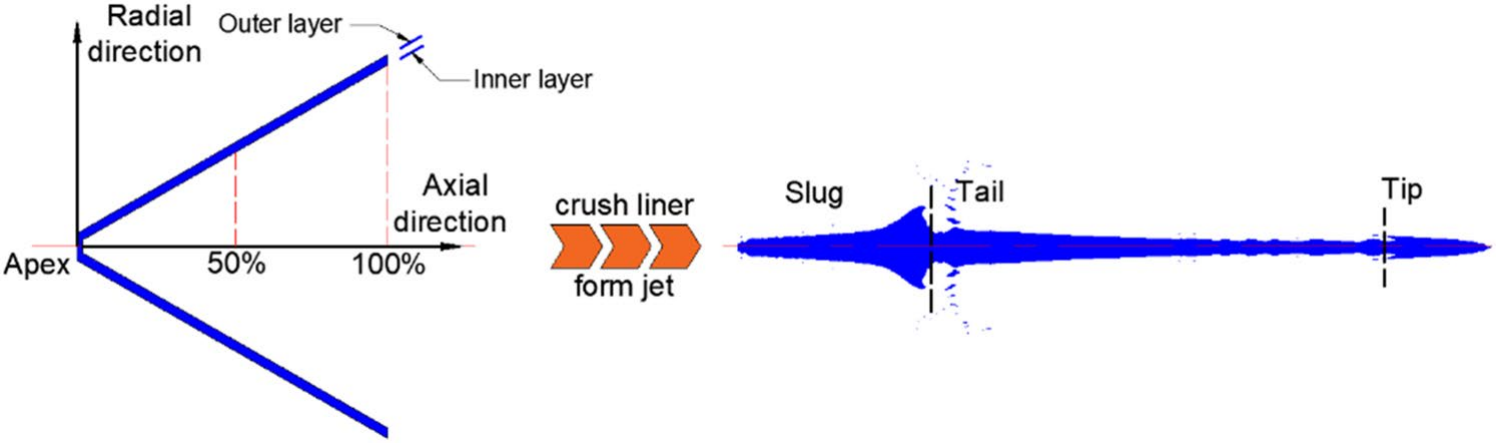
Schematic of the position of the liner and the jet.

To understand the characteristics of particle motion in different layers of the liner, several mass points were set. The velocities and displacements of the particles at different positions of the liner were measured and plotted, as shown in Fig. 13. The position of the liner and the jet is shown in Fig. 14.

The results show that variations in the axial velocities of the outer elements of Zr and Vit1 were basically the same, and both materials formed the jet tail or slug. In contrast, a considerable part of the inner particles formed the jet tip and its main body. To understand this phenomenon, the movement of the particles during the crushing process of the liner was further analyzed.

**Figure 15:**
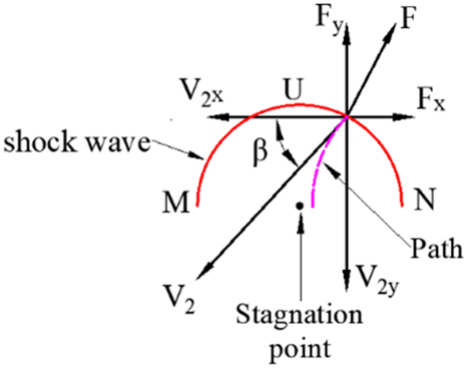
Velocity and force of the liner in the moving coordinate system.

Extremely high pressure was generated at the collision point, and a shock wave was formed. The outward propagating shock wave and the material that accumulated near the collision point affected the subsequent motion of the particles. When the particle was affected, flow velocity $V_{2}$ and force *F* were decomposed in the moving coordinate system, as shown in Fig. 15.

Since the position where the particle interacted with the shock wave was always located on the curve $MUN$ (relative to stagnation point),$F_{y}$ finally reduced $V_{2y}$ to a very low level; the change in $V_{2x}$ in the horizontal direction was related to the position of the action point. In the *UN* interval, $F_{x}$ hindered the horizontal movement of particles so that $V_{2x}$ continued to decrease. When the particles were in the *UM* segment, $F_{x}$ facilitated the movement of the particle. For $V_{2}$, $F$ and the collapse angle $\beta$ of the particles constantly changed with the movement, and the motion trajectory of the particle was shown as a curve.

**Figure 16:**
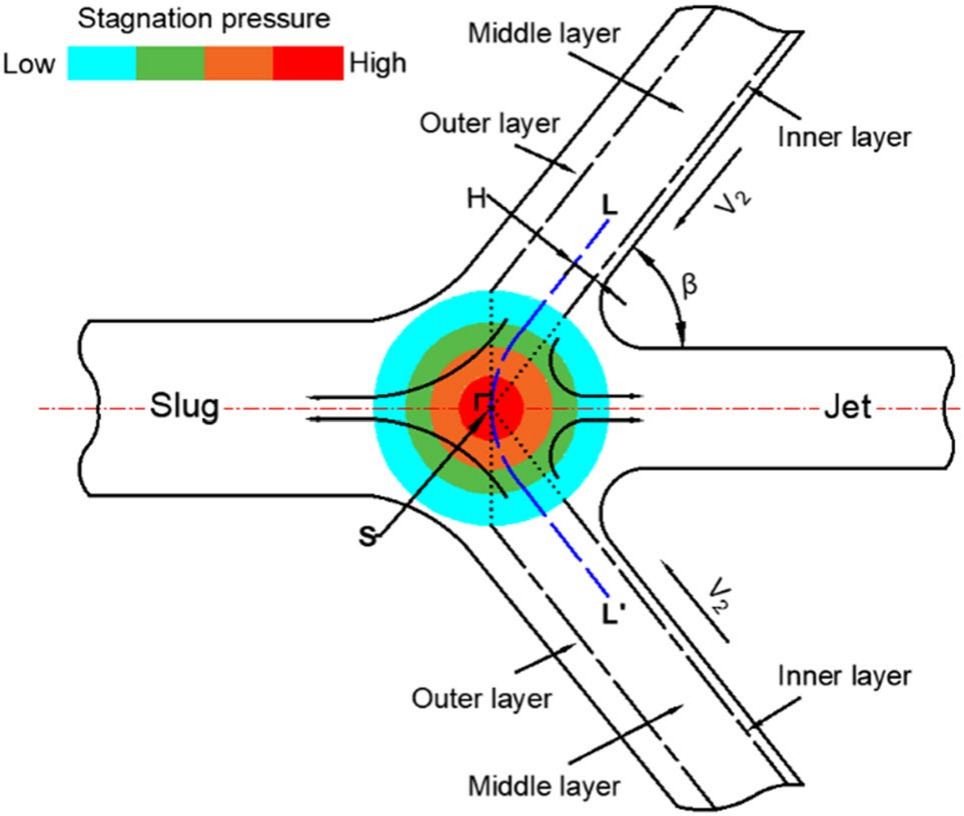
Schematic of particle motion.

According to the motion of the particles shown in Fig. 15, the liner can be finely divided into three layers, as shown in Fig. 16. Under the influence of the shock wave, the particles in the outer layer moved in the direction of the slug, while the particles in the inner layer formed a jet. For the middle layer, the situation was slightly complicated. Briefly, there was a particle motion trajectory LSL' called the “escape limit,” which was related to $V_{2}$, $\beta$, and *F*. In the figure, *H* represents the thickness of the liner before the escape limit. The particles behind the escape limit clearly moved in the direction of the slug, while the other particles were prone to form the jet. High-speed particles generated strong shock waves, which caused the particle’s $V_{2x}$ to decay rapidly. A large $\beta$ decreased the particle’s $V_{2x}$ and rapidly decreased the particle’s axial velocity. Both of these conditions led to an increase in *H*, which enabled more high-velocity particles to form the jet head or its main body. This phenomenon explains the difference in the motion of the different layer particles of the liner shown in Fig. 13.

### Particles near the top of the liner

**Figure 17:**
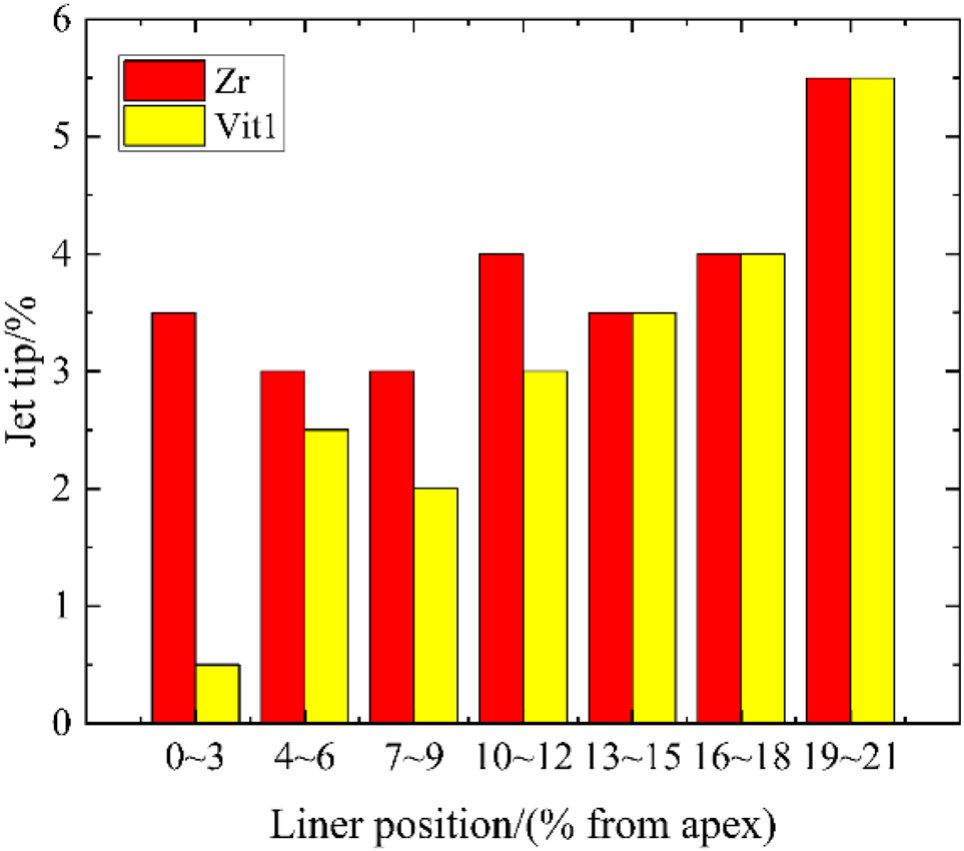
Proportion of the jet tip formed by particles near the top of the liner.

The velocity and displacement of the inner layer particles near the top of the two liner materials were obviously different, as shown in Fig. 13. Multiple mass points were set in the inner layer thickness of 0.3 mm near the top of the liner, and the proportion of jet tip formed is shown in Fig. 17.

In the range of approximately 10%, the proportion of the inner layer particles of Zr forming the jet tip was relatively stable. However, the proportion of the inner layer particles of Vit1 forming the jet tip was significantly lower than that of Zr and was only ~ 0.5% within ~ 3% of the liner apex. With increasing distance from the liner apex, the proportion of the inner layer particles forming the jet tip of the two materials increased rapidly and showed highly consistent numerical values.

According to the unsteady state Pugh–Eichelberger–Rostoker (PER) model^30,31^, collapse angle $\beta$ is negatively correlated with flow velocity $V_{2}$. The collapse angle of the particles near the top is small, and velocity $V_{2}$ is large. Figure 16 shows that the middle layer particles near the apex could easily cross the escape limit and move toward the slug.

**Figure 18:**
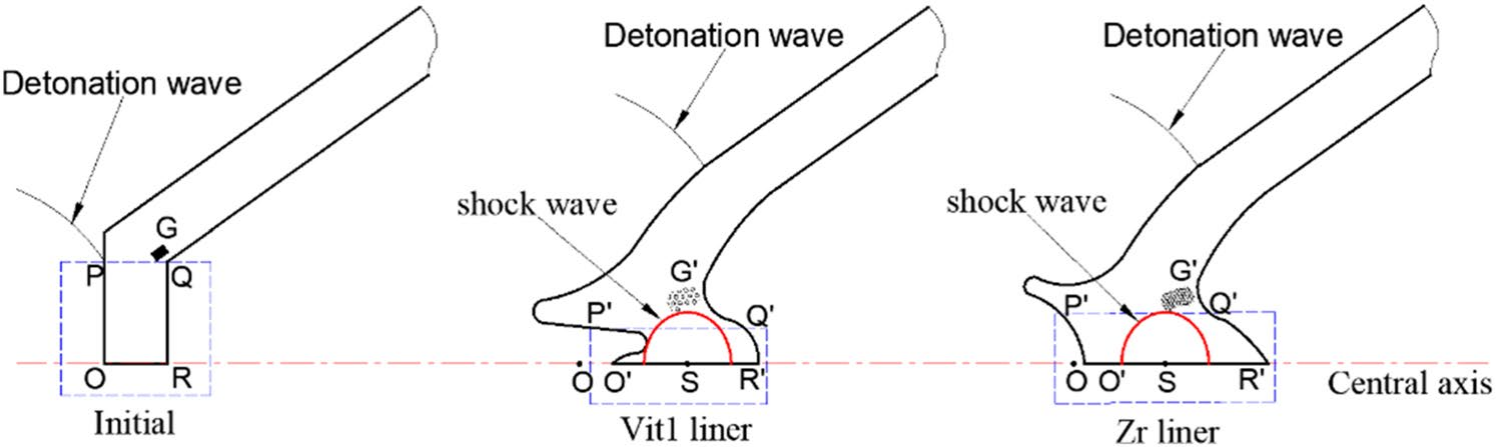
Schematic of the deformation of the two material liners.

In addition, the crushing process at the top of the liner was carefully observed using the numerical method, and the schematic is shown in Fig. 18. The truncated part OPQR of the liner first underwent shear fracture and accelerated the forward movement under the impact of the detonation wave. The truncated part deformed and moved to the O'P'Q'R' position with time, while particle set G moved to the G' position. The difference is in the relationship between particle set G' and stagnation point S.

**Figure 19:**
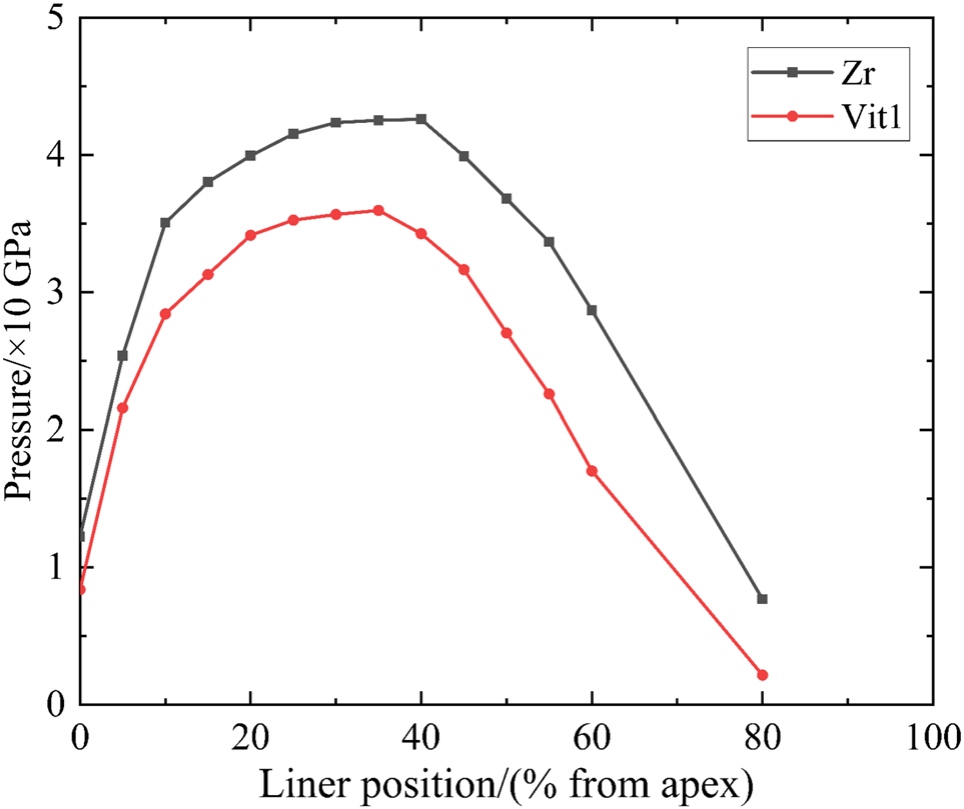
Stagnation pressure curve.

Studies have shown that amorphous alloy materials have long-range disordered and short-range ordered microstructures and exhibit no dislocation slip behavior compared with crystalline materials^32^. The materials generally exhibit typical brittle fracture characteristics under high strain rates^33–36^, with a low shear strength. Vit1 is more prone to fracture than Zr and moves forward to create a large OS distance, as shown in Fig. 18. In addition, particle set D showed a loose particle cluster structure, in which the bonding force between particles was extremely small^37,38^. The corresponding shock wave pressure was also low because the stagnation pressure was small when the Vit1 liner collapsed (Fig. 19). Therefore, on the one hand, particle set G' of Vit1 was more backward than that of Zr relative to stagnation point S; on the other hand, force *F* (as shown in Fig. 15) on the particles of the Vit1 liner was also small. Consequently, the particles near the liner apex of Vit1 could easily cross the escape limit but hardly form a jet.

The study of particle motion at different positions of the liner revealed that force *F* had a significant influence on particle motion. Clearly, the magnitude of *F* was related to the shock wave intensity, which was directly related to the collision pressure. A comparison of Figs. 13 and 19 showed that the pressure and the axial movement trend of the particles were similar. Therefore, theoretical analysis was performed to clarify the relationship between the pressure and the motion of the particles.

## Theoretical model analysis

**Figure 20:**
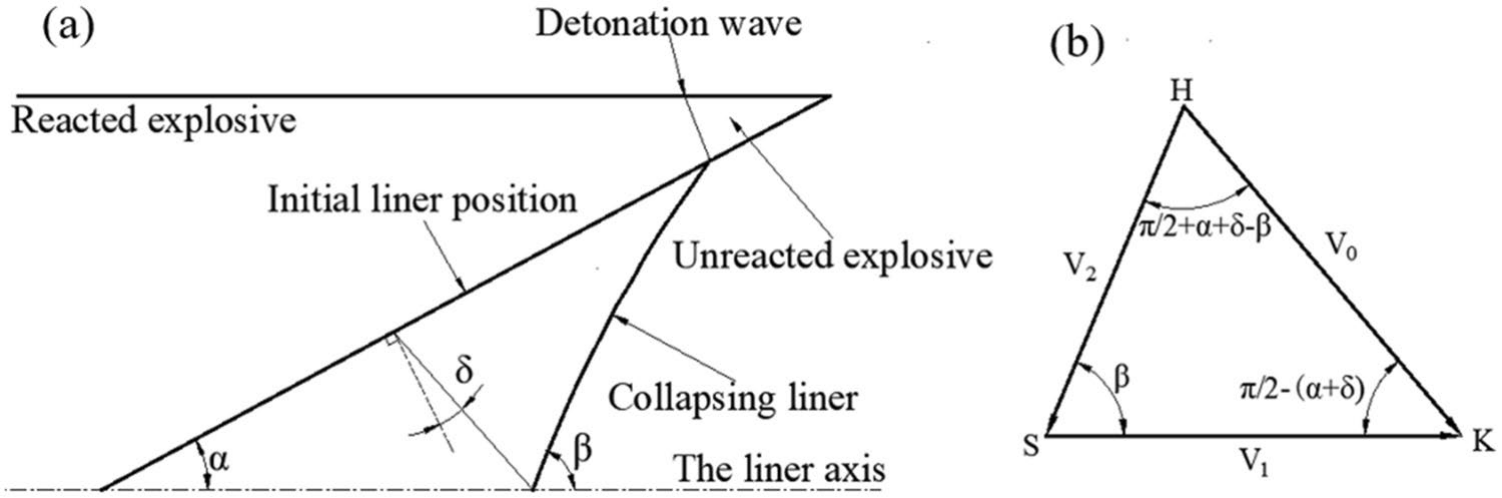
(**a**) Schematic of the collapse process; (**b**) velocity vectors in a moving system.

Pugh et al. modified the theory of steady-state ideal incompressible fluid mechanics^39–41^ and proposed PER theory^30^, in which the collapse velocity of the shaped charge liner varies. The collapse velocity gradually decreased from the apex to the bottom so that the surface of the liner no longer remained straight during the collapse, as shown in Fig. 20a.

A moving coordinate system was established at stagnation point S, and the velocity relationship is shown in Fig. 20b.

The calculation equations can be obtained from the geometric relationship as follows:

1 $$ V_{1} = \frac{{V_{0} \cos (\beta - \alpha - \delta )}}{\sin \beta };\quad V_{2} = \frac{{V_{0} \cos (\alpha + \delta )}}{\sin \beta } $$

where $V_{0}$ is the collapse velocity, $V_{1}$ is the stagnation velocity, $2\alpha$ is the cone angle, and $\delta$ is the deflection angle. On the basis of the PER model, the deflection angle is calculated as follows:

2 $$ \delta = \sin^{ - 1} \left( {\frac{{V_{0} }}{2U}} \right);\quad U = U_{D} /\cos \alpha $$

where $U_{D}$ is the detonation velocity.

Thus, the velocities of the jet ($V_{j}$) and slug ($V_{s}$) in the static coordinate system are calculated as follows:

3 $$ V_{j} = V_{1} + V_{2} ;\quad V_{s} = V_{1} - V_{2} $$

According to PER theory, scientists have carried out further research on jet formation. Chou et al. ^1^ proposed the criteria for jet formation on the basis of experimental and theoretical studies and believed that the cohesion of the jet was related to the sound speed of the liner material, the flow velocity ($V_{2}$) of the jet, and the collapse angle $\beta$, as shown in Table 7.

**Table 7:** Conditions for jet formation and coherency.

Flow regime	Collapse angle *β*	Jet formation	Jet coherence
Supersonic ($V_{2} > C_{L}$)	$\beta \le \beta_{c}$	No	No
	$\beta > \beta_{c}$	Yes	No
Subsonic ($V_{2} \le C_{L}$)	All values	Yes	Yes

The magnitude of flow velocity $V_{2}$ is a prerequisite for jet cohesion; specifically, only under the premise of supersonic motion can a noncohesive jet be formed when collapse angle $\beta$ is greater than the critical value. However, $\beta$ is difficult to obtain accurately, restricting the application of relevant theories. Therefore, a model for predicting particle motion and jet forming, which can avoid the frequent use of $\beta$, is proposed.

When the sound speed ($C_{L}$) of the material is known, the critical collapse angle $\beta_{CL}$ can be obtained from Eq. (1):

4 $$ \beta_{cL} = \sin^{ - 1} \frac{{V_{0} \cos (\alpha + \delta )}}{{C_{L} }} $$

In related studies^42,43^, collapse angle $\beta$ can also be obtained by using momentum conservation.

5 $$ \tan^{2} \beta = \frac{{P\left[ {\rho_{0} V_{2}^{2} (\frac{\mu }{\mu + 1}) - P} \right]}}{{(\rho_{0} V_{2}^{2} - P)^{2} }} $$

where $P$ is the pressure.

Therefore, the pressure threshold ($P_{CL}$) corresponding to $\beta_{CL}$ can be calculated from Eq. (5).

6 $$ P_{cL} = \frac{{\left[ {2(\mu + 1)\tan^{2} \beta_{cL} + \mu + \sqrt {\mu^{2} - 4\mu \tan^{2} \beta_{cL} - 4\tan^{2} \beta_{cL} } } \right]\rho_{0} C_{L}^{2} }}{{2(\mu + 1)(1 + \tan^{2} \beta_{cL} )}} $$

Substituting Eq. (4) into Eq. (6) would yield the following equation:

7 $$ P_{cL} = \frac{{\left[ {2(\mu + 1)\tan^{2} \left( {\sin^{ - 1} \frac{{V_{0} \cos (\alpha + \delta )}}{{C_{L} }}} \right) + \mu + \sqrt {\mu^{2} - 4(\mu + 1)\tan^{2} \left( {\sin^{ - 1} \frac{{V_{0} \cos (\alpha + \delta )}}{{C_{L} }}} \right)} } \right]\rho_{0} C_{L}^{2} }}{{2(\mu + 1)\left[ {1 + \tan^{2} \left( {\sin^{ - 1} \frac{{V_{0} \cos (\alpha + \delta )}}{{C_{L} }}} \right)} \right]}} $$

where $\rho_{0}$ is the initial liner density and $\mu$ is the compression ratio ($\mu = \rho /\rho_{0} - 1$).

In addition, critical collapse angle $\beta_{c}$ can be determined from Eq. (5) with condition $d\beta /d\mu = 0$^43^. Thus, for $\beta = \beta_{c}$, the equation is as follows:

8 $$ \frac{{{\text{d}}P}}{{{\text{d}}\mu }} = \frac{{P[P - \rho_{0} V_{2}^{2} ]}}{{(\mu + 1)[\mu \rho_{0} V_{2}^{2} - P(\mu + 2)]}} $$

The critical compression ratio ($\mu_{c}$) can be calculated by combining the equation of state (EOS) of the material and Eq. (8).

For conventional elastoplastic metal materials, such as Zr, shock EOS is usually used. The corresponding equations have been clarified in the literature^28^ and are not described here. The improved Johnson–Cook model (JH-2) is considered highly suitable for brittle materials, such as Vit1^44–47^. The model can be expressed as follows:

9 $$ P = K_{1} \mu + K_{2} \mu^{2} + K_{3} \mu^{3} $$

where $K_{1}$, $K_{2}$, and $K_{3}$ are the parameters related to the material, and $K_{1}$ is usually the bulk modulus.

According to Eq. (9), the following relationship exists:

10 $$ \frac{{{\text{d}}P}}{{{\text{d}}\mu }} = K_{1} + 2K_{2} \mu + 3K_{3} \mu^{2} $$

The combination of Eqs. (8) and (10) yields the following:

11 $$ \frac{{P[P - \rho_{0} V_{2}^{2} ]}}{{(\mu + 1)[\mu \rho_{0} V_{2}^{2} - P(\mu + 2)]}} = K_{1} + 2K_{2} \mu + 3K_{3} \mu^{2} $$

If we allow Eq. (10) = *X*; the following relationship is obtained:

12 $$ P^{2} + [X(\mu + 1)(\mu + 2) - \rho_{0} V_{2}^{2} ]P - X\mu (\mu + 1)\rho_{0} V_{2}^{2} = 0 $$

13 $$ P_{c} = \frac{{\rho_{0} V_{2}^{2} - X(\mu + 1)(\mu + 2) \pm \sqrt {[X(\mu + 1)(\mu + 2) - \rho_{0} V_{2}^{2} ]^{2} + 4X\mu (\mu + 1)\rho_{0} V_{2}^{2} } }}{2} $$

From the JH-2 model, we obtain the following:

14 $$ K_{1} \mu + K_{2} \mu^{2} + K_{3} \mu^{3} = \frac{{\rho_{0} V_{2}^{2} - X(\mu + 1)(\mu + 2) \pm \sqrt {[X(\mu + 1)(\mu + 2) - \rho_{0} V_{2}^{2} ]^{2} + 4X\mu (\mu + 1)\rho_{0} V_{2}^{2} } }}{2} $$

If we set Eq. (9) = *Y* and $X(\mu + 1)(\mu + 2) - \rho_{0} V_{2}^{2} = W$; thus, the following equations are obtained:

15 $$ 2Y + W = \pm \sqrt {W^{2} + 4X\mu (\mu + 1)\rho_{0} V_{2}^{2} } $$

16 $$ Y^{2} + YW = X\mu (\mu + 1)\rho_{0} V_{2}^{2} $$

The values of *X*, *Y*, and *W* are substituted to calculate $\mu_{c}$. The degree of $\mu$ in the equation is high, and the simulation results show that $\mu$ is always in the [0,1] interval. Therefore, Eq. (16) can be simplified as follows:

17 $$ \begin{aligned} & [K_{1}^{2} + 4K_{2}^{2} + 11K_{1} K_{2} + 8K_{1} K_{3} - 2K_{2} - (3 + \rho_{0} V_{2}^{2} )K_{3} ]\mu^{3} \\ & \quad + [4K_{1}^{2} + 6K_{1} K_{2} - K_{1} - (2 + \rho_{0} V_{2}^{2} )K_{2} ]\mu^{2} + [2K_{1}^{2} - (1 + \rho_{0} V_{2}^{2} )K_{1} ]\mu = 0 \\ \end{aligned} $$

During the crushing process of the liner, $\mu \ne 0$; thus, the following equation is obtained:

18 $$ \begin{aligned} & [K_{1}^{2} + 4K_{2}^{2} + 11K_{1} K_{2} + 8K_{1} K_{3} - 2K_{2} - (3 + \rho_{0} V_{2}^{2} )K_{3} ]\mu^{2} \\ & \quad + [4K_{1}^{2} + 6K_{1} K_{2} - K_{1} - (2 + \rho_{0} V_{2}^{2} )K_{2} ]\mu + 2K_{1}^{2} - (1 + \rho_{0} V_{2}^{2} )K_{1} = 0 \\ \end{aligned} $$

The critical compression ratio ($\mu_{c}$) at a given flow velocity ($V_{2}$) can be obtained from Eq. (18). Then, critical pressure $P_{c}$ and critical collapse angle $\beta_{c}$ can be obtained from Eqs. (9) and (5), respectively.

By comparing the stagnation pressure ($P_{t}$) of the different positions of the liner and pressure threshold $P_{CL}$, critical pressure $P_{c}$ and the axial and radial movement trend of the particles can be predicted, as shown in Table 8.

**Table 8:** Conditions for the motion trend of particles.

Pressure *P*_t_	Axial trend	Pressure *P*_t_	Radial trend
$P_{t} > P_{CL}$	Tip	$P_{t} < P_{c}$	Scatter
		$P_{t} \ge P_{c}$	Converge
$P_{t} \le P_{CL}$	Tail	All values	Converge

**Figure 21:**
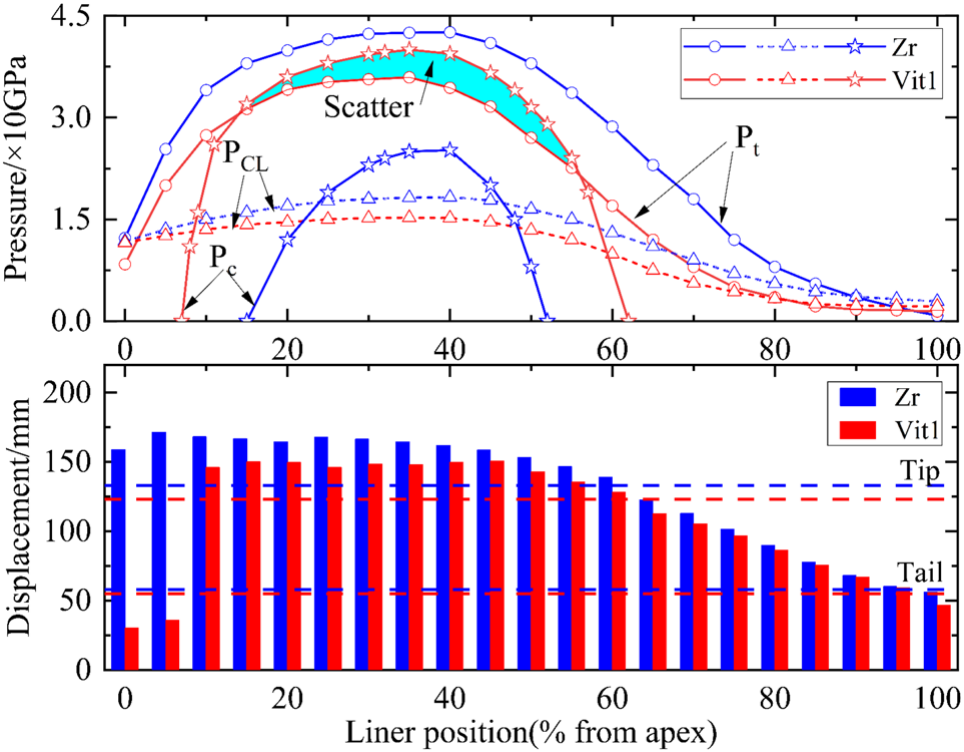
Verification curve of inner particle motion.

Stagnation pressure $P_{t}$ can be easily obtained through numerical simulations. The two liner materials involved in this work verified the relevant conclusions, and the results are shown in Fig. 21. When $P_{t}$ was greater than $P_{CL}$, the particles predominantly formed the jet tip or approached the jet head. Part $P_{c}$ of Vit1 was larger than $P_{t}$, indicating that many particles dispersed radially. This result was consistent with the experimental results. In addition, Zr formed a cohesive jet because its $P_{c}$ was always lower than $P_{t}$, which was consistent with the related research results ^28^.

Table 8 presents a novel method of evaluating jet cohesiveness and predicting particle motion at different positions of the liner and avoids the frequent use of the collapse angle, which is difficult to accurately obtain. For a noncohesive jet, the range of radial discrete particles generated by the liner can be obtained. The radial motion trend of the particles in a specific region can be predicted.

## Conclusions

X-ray experiments were performed on a Zr-based amorphous alloy jet. The results showed that the alloy formed a noncohesive jet driven by the explosion of an 8701 explosive. The jet simulation results confirmed the applicability of the JH-2 model for Zr-based amorphous alloys. The comparison of different numerical simulation algorithms indicated that both the Euler and SPH algorithms could ensure highly accurate calculations of jet velocity and length. Within a short computing time, the Euler algorithm effectively reflected the jet shape, while the SPH algorithm was suitable for representing the jet tail for a long calculation time. The flared mouth in the 3D model using the Euler algorithm indicated the radial divergence of the jet; however, the radial divergence could be directly observed in the 2D model when the JH-2 material model was used. The simulation of the Zr and Zr-based amorphous alloy jets revealed that the fracture properties of the material had an effect on the proportion of particles near the top of the conical liner for forming jet; a more brittle material correlated to a lower proportion of particles. The movement and force of the particles during the collapsing process were qualitatively analyzed, and the boundary at which the particles could or could not form a jet was determined. Finally, due to the difficulty of determining the collapse angle, a new method was proposed in which pressure was used to enable the prediction of the movement trend of particles.

## Supplementary Information

Supplementary Information.

## Data Availability

All data generated or analysed during this study are included in this published article and its supplementary information files.
