# Supplementary material for: Research on non-cohesive jet formed by Zr-based amorphous alloys
Source: Sci Rep. 2023 Mar 13;13:4149. doi: 10.1038/s41598-023-30836-0 (PMC10011371; doi:10.1038/s41598-023-30836-0)
Supplement: Supplementary file 1 — Supplementary Information. [file 41598_2023_30836_MOESM1_ESM.docx]

**Appendix**

In the jet formation simulation, a suitable material model must be adopted. The experimental study revealed that the JH-2 model is suitable for Zr-based amorphous alloy materials and can be used to characterize their unique mechanical properties.

The EOS for the JH-2 model can be described as follows:

$P=K_{1}\mu+K_{2}\mu^{2}+K_{3}\mu^{3}$ (A1)

where $K_{1}$, $K_{2}$, and $K_{3}$ are the parameters related to the material, and $\mu$ is the compression ratio. $K_{1}$ usually takes the bulk modulus.

The equivalent stress ($\sigma^{*}$) of the material is expressed as a power function of the hydrostatic pressure, which is related to the strain rate and damage factor $D$.

$\sigma^{*}=\sigma_{i}^{*}-D(\sigma_{i}^{*}-\sigma_{f}^{*})$ (A2)

where $\sigma_{i}^{*}$ is the normalized complete strength of the material, and $\sigma_{f}^{*}$ is the normalized fracture strength of the material.

When the material is not damaged, the following relationship is obtained:

(A3)

When the material is completely damaged, the following relationship is obtained:

(A4)

The damage model in the JH-2 constitutive model uses the form of cumulative damage, as follows:

$D=\sum(\Delta\varepsilon_{p}/\varepsilon_{p}^{f})$ (A5)

where $\Delta\varepsilon_{p}$ is the equivalent plastic strain of the material, and $\varepsilon_{p}^{f}=f(p)$ is the plastic strain of the material breaking under constant pressure $P$.

$\varepsilon_{p}^{f}=D_{1}(p^{*}+T^{*})^{D_{2}}$ (A6)

where $D_{1}$ and $D_{2}$ are material constants.

The JH-2 model parameters of Zr-based amorphous alloys can be obtained by fitting and iterating the mechanical property test data, as shown in Table A1.

Table A1 JH-2 model parameters of Vit1 [47]

| *K*_1_/GPa | *K*_2_/GPa | *K*_3_/GPa | *D*_1_ | *D*_2_ |
| --- | --- | --- | --- | --- |
| 114.3 | 268.5 | 1386 | 0.21 | 1.75 |
| *A* | *B* | *C* | *M* | *N* |
| 1.162 | 0.258 | 0.0173 | 0.59 | 0.829 |

In addition, the data in the Autodyn material library were used as the Zr material parameters.

The 8701 explosive is composed of hexogen (RDX), polyvinyl acetate (PVAC), dinitrotoluene (2,4-DNT), and calcium stearate (CaSt). The EOS for the 8701 explosive is the JWL model.

$P=A(1-\frac{\omega}{r_{1}v})e^{-r_{1}v}+B(1-\frac{\omega}{r_{2}v})e^{-r_{2}v}+\frac{\omega E}{v}$ (A7)

where $P$ is the pressure, $v=1/\rho$is the specific volume, $\rho$ is the current material density, $E$ is the specific internal energy per unit mass, and $A$, $B$, $r_{1}$, $r_{2}$, and $\omega$ are material constants. Parameters are obtained by experiments and data fitting and are listed in Table A2 (*D*_e_ and *P*_CJ_ are detonation velocity and detonation wave C-J pressure, respectively).

Table A2 Parameters of the 8701 explosive

| *ρ*/g·cm^-3^ | *A*/kPa | *B*/kPa | *r*_1_ | *r*_2_ | $\omega$ | *D*_e_/m·s^-1^ | *E*/kJ·m^-3^ | *P*_CJ_/kPa |
| --- | --- | --- | --- | --- | --- | --- | --- | --- |
| 1.72 | 6.184×10^8^ | 6.900×10^6^ | 4.3 | 0.87 | 0.38 | 8350 | 8.5×10^6^ | 2.966×10^7^ |
